# Cellulose-Based Absorbent Production from Bacterial Cellulose and Acrylic Acid: Synthesis and Performance

**DOI:** 10.3390/polym10070702

**Published:** 2018-06-25

**Authors:** Mu-Tan Luo, Hai-Long Li, Chao Huang, Hai-Rong Zhang, Lian Xiong, Xue-Fang Chen, Xin-De Chen

**Affiliations:** 1 Key Laboratory of Renewable Energy, Chinese Academy of Sciences, Guangzhou 510640, China; mutan.l@hotmail.com (M.-T.L.); lihl@ms.giec.ac.cn (H.-L.L.); zhanghr@ms.giec.ac.cn (H.-R.Z.); xionglian@ms.giec.ac.cn (L.X.); chenxue_228@126.com (X.-F.C.); 2 Guangzhou Institute of Energy Conversion, Chinese Academy of Sciences, Guangzhou 510640, China; 3 Guangdong Provincial Key Laboratory of New and Renewable Energy Research and Development, Guangzhou 510640, China; 4 University of Chinese Academy of Sciences, Beijing 100049, China; 5 Xuyi Center of Attapulgite Research Development & Industrialization, Guangzhou Institute of Energy Conversion, Chinese Academy of Sciences, Xuyi 211700, China

**Keywords:** bacterial cellulose, superabsorbent, polymerization, function and structure

## Abstract

Cellulose-based superabsorbent was synthesized by bacterial cellulose (BC) grafting acrylic acid (AA) in the presence of *N*,*N*′-methylenebisacrylamide (NMBA) as a crosslinker and ammonium persulfate (APS) as an initiator. The influence of different factors on composite synthesis, including the weight ratio of the monomer to BC, initiator content, crosslinker content, AA neutralization degree, reaction temperature, and reaction time on the water absorbency of the composite, were systematically learned. Under the optimized conditions, the maximum water absorbency of the composite was 322 ± 23 g/g distilled water. However, the water absorbency was much less for the different salt solutions and the absorption capacity of the composite decreased as the concentration of the salt solutions increased. The pH value had a significant influence on water absorption performance, and with the increase of temperature, the water retention rate of the composite decreased. Additionally, the structure of this composite was characterized with nuclear magnetic resonance (NMR), Fourier transform infrared spectroscopy (FT-IR), scanning electron microscopy (SEM), and thermogravimetric analysis (TGA). The results of NMR and FT-IR provided evidence that the composite was synthesized by BC and AA, and the microstructure showed that it had good performance for water absorption. In addition, the composite possessed suitable thermal stability, and that it could be used in a few high-temperature environments. Overall, this composite is promising for application in water absorption.

## 1. Introduction

Synthesis of superabsorbent composite is a hot topic in the field of materials [1,2,3]. Since there exists a weakly-crosslinked three-dimensional network structure in the superabsorbent material, the material has the ability to absorb considerable amounts of water, or other liquids, in a relatively short time [4], thus, it can be applied in many fields, such as agricultural and forestry industries, environmental protection, biomedical application, and so on [5,6,7,8,9,10,11,12]. Starch-based superabsorbent composite has been applied in modern society, and this superabsorbent composite is the product of starch grafted with some substances which contain hydrophilic groups, such as acrylic acid, acrylonitrile, acrylamide, maleic anhydride, and so on [13,14]. However, starch-based superabsorbent is imperfect, and it also has some defects. The most serious disadvantage is that the raw material of traditional superabsorbent composite is starch, and this creates the “food vs. material” debate which will undoubtedly limit the industrialization of this material. Therefore, how to overcome the shortcoming and improve the performance of traditional superabsorbent composites is one of the most important studies in the related fields.

In order to solve the “food vs. material” debate that is induced by starch-based superabsorbent composites, other materials have been used in the production of superabsorbent composites. Cellulose is the most abundant organic material in the world. It is well known that it has outstanding renewable and biodegradable properties, and it is not only biodegradable, but also contains many hydroxyl groups, so it has strong water absorption [15]. In addition, because cellulose is not a grain, it cannot lead to the “food vs. material” conflict that starch causes. However, compared to microbial cellulose, which contains the single cellulose form, the purity and strength of plant cellulose is relatively low due to the existence of hemicellulose and lignin in its composition, in addition to cellulose [16]. Obviously, this is a disadvantage for the practical applications of materials. In contrast, microbial cellulose, rather than plant cellulose, with high purity and strength becomes an adequate ingredient of water absorbent synthesis.

Bacterial cellulose (BC) is a renewable and abundant biomaterial (polysaccharide) containing long non-aggregated nanofibrils usually generated by *Acetobacter xylinum* [17]. In the process of BC production, the main carbon sources are generally glucose, mannitol, sucrose, etc., and sometimes a low-cost feedstock such as lignocellulosic biomass [18]. Although the yield of BC varies according to the different carbon sources and production conditions, its availability is somehow comparable to plant cellulose because of its simple and easy production requirements. Since it has excellent structural and biochemical properties, such as an ultra-fine highly-pure nanofibril network structure (its crystallinity is up to 95%, while plant cellulose’s crystallinity is only 65%), high tensile strength (in general, the strength of bacterial cellulose is more than ten times higher than that of plant cellulose), adaptability to the living body, biodegradability, chemical stability, and non-toxicity, especially because of its special network structure, it has a strong capability of water absorption and binding, and it has a wide range of applications, including food, biomedical material, papermaking, and transducer diaphragms [19,20,21,22,23,24,25,26,27]. Owing to the higher capability of water absorption, BC is widely used as a new raw material for superabsorbent composite synthesis. However, with the development of modern industry, the absorption capability of raw BC cannot satisfy the needs of the application, therefore, its absorption capacity must be further improved. 

Usually, BC can be modified to optimize its performance, and the most common way is graft copolymerization (acrylic acid (AA) is applied commonly as the substrate in this reaction) [28,29,30,31]. The absorbent material synthesized by BC and AA are generally polymer electrolytes containing hydrophilic groups and cross-linked structures. The mechanism for water absorption by this composite has been initially elucidated. Generally, before absorbing water, the polymer chains are closely linked and linked to each other to form a network structure, so as to achieve overall fastening. When contacted with water, water molecules penetrate into the composite through capillary action and diffusion, and the ionization groups on the chain ionize in water. Due to the electrostatic repulsion between the ions on the chain, the polymer chain expands and swells. Due to the electrical neutrality requirement, counter ions cannot migrate to the outside of the composite, and the ion concentration difference between the inside and outside of the composite forms reverse osmosis. Then, water enters the composite further under the action of reverse osmosis and forms hydrogels. Due to these types of chemical compositions and structures, the composite possesses strong water absorption capacity.

**Figure 1:**
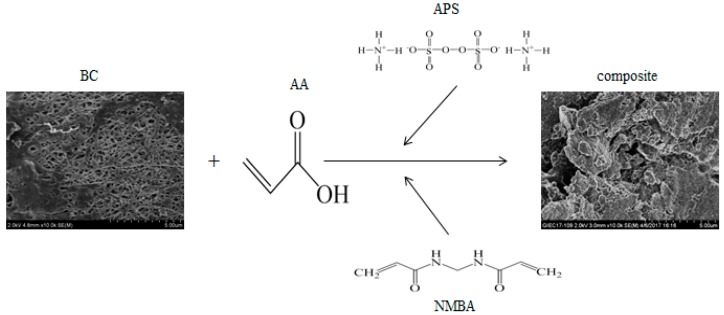
Reaction scheme for cellulose-based absorbent production.

In this study, BC was grafted with an effective monomer (AA) to synthesize a new hydrogel composite (the scheme for synthesis is shown in Figure 1). In detail, the influence of different factors on composite synthesis, such as the weight ratio of monomer to BC, initiator content, crosslinker content, AA neutralization degree, reaction temperature, and reaction time on the water absorbency of the composite, were systematically learned. Meanwhile, the composite obtained after this reaction was characterized by several methods, including nuclear magnetic resonance (NMR), Fourier transformed infrared spectroscopy (FT-IR), scanning electron microscopy (SEM), and thermogravimetric analysis (TGA). The capacities of water absorbency, different salt solution absorbency, absorbency at various pH values, and water retention at various temperatures were also analyzed, respectively. 

## 2. Materials and Methods

### 2.1. Materials

BC was obtained from Hainan Yide Co., Ltd. (Hainan, China), and *Gluconacetobacter xylinus* is the bacterium used for BC production. Before use, the BC was ground into powder. Cyclohexane (analytical grade) was bought from Tianjin Fuyu Fine Chemical Co., Ltd. (Tianjin, China), acrylic acid (analytical grade, Tianjin Fuchen Chemical Reagent Factory, Tianjin, China) was neutralized by NaOH (analytical grade, Guangzhou Chemical Reagent Factory, Guangzhou, China) before use, ammonium persulfate (analytical grade, Sino Pharm Chemical Reagent Co., Ltd., Beijing, China) was dissolved in water before use, *N*,*N*′-methylenebisacrylamide (analytical grade, Tianjin Kemiou Chemical Reagent Factory, Tianjin, China) was dissolved in dimethyl sulfoxide (analytical grade, Aladdin Industrial Co., Ltd., Shanghai, China) before use.

### 2.2. Graft BC by AA

BC (1 g) was transferred into a 250 mL four-neck flask, 100 mL cyclohexane was added into the flask, and then kept stirring in nitrogen atmosphere for thirty minutes. After that, ammonium persulfate (APS) solution and *N*,*N*′-methylenebisacrylamide (NMBA) solution were mixed into the neutralized AA. The content range of APS and NMBA were 1–5% and 0–0.4% (*w*/*w*) of the monomer, respectively, and the neutralization range of AA was 70–90%. Meanwhile, the ratio of BC to AA was from 1:3 to 1:8. Then the mixture was added into the above BC-cyclohexane mixed system drop by drop with a peristaltic pump (5 rpm). The reaction completed under the condition of the reaction temperature range from 55 to 75 °C after 0.5–4 h by maintaining the nitrogen atmosphere throughout the whole process. After the reaction, the composite was washed with distilled water firstly, and then the composite was further stirred in ethanol for 1 h to eliminate the impurities (by-products from acrylic acid self-polymerization, unreacted monomer, residual APS, and NMBA, etc.) possibly existing in the product. Finally, the composite was washed with distilled water again, and then separated from water and dried to constant weight by lyophilization.

### 2.3. Characterizations

#### 2.3.1. Nuclear Magnetic Resonance

The structure of the superabsorbent composite was tested by ^13^C NMR (solid state), using a Brook’s AVANCE type III nuclear magnetic resonance spectrometer (Karlsruhe, Germany), and the chemical shift zero point was calibrated with tetramethylsilane.

#### 2.3.2. Fourier Transformed Infrared Spectroscopy

Fourier transform infrared spectroscopy (FT-IR) was performed on a FT-IR spectrometer (Bruker Corporation, Tensor 27, Karlsruhe, Germany), and the spectra was obtained between 4000 and 400 cm^−1^. The composite was dispersed in dry KBr, and then the composite with KBr was analyzed by FT-IR.

#### 2.3.3. Scanning Electron Microscope

The physical surface morphology of superabsorbent composite was observed with a Hitachi SU70 scanning electron microscope (Hitachi, Tokyo, Japan) operated at 2.0 kV and 10 μA. The sample was sprayed with gold powder before operation, and then photographed. 

#### 2.3.4. Thermogravimetric Analysis

In thermogravimetric analysis, the composite was detected by a thermogravimetric analyzer (NETZSCH, STA409C-GC2014, Bavaria, Germany). In detail, the superabsorbent composite (approximately 10 mg) was placed in an aluminum crucible, and the heating temperature was from room temperature to 650 °C at the rate of 10 °C min^−1^ under a nitrogen purge. The weight loss (TG curve) and the differential thermogravimetric (DTG) curve were recorded simultaneously. 

#### 2.3.5. Performance of Absorption

##### The Capacity of Water Absorbency

The water absorbency capacity of the synthesized composite was determined by gravimetric analysis. For measurement, the sample (approximate 0.1 g) was immersed in distilled water (100 mL) at room temperature for 12 h to reach swelling equilibrium. Then, the swollen sample was separated from the unabsorbed water by filtration through an 80-mesh nylon bag until no free water dripped down. The water absorbency of composite (*Q*_eq_) was calculated using the following equation:

(1) $$\;Q_{\mathrm{eq}}=\frac{w_{2}-w_{1}}{w_{1}}$$

where *Q*_eq_ is the water absorbency per gram of dried sample (g/g), and *w*_1_ and *w*_2_ are the mass weights of the dry and swollen samples (g), respectively. 

In addition, water absorbency capacity was also measured in salt solutions (NaCl, KCl, NH_4_Cl, CuCl_2_, and CaCl_2_) with different concentrations (0%, 0.1%, 0.3%, 0.6%, 0.9%, 1.2%) at the equilibrium swelling state. Additionally, water absorbency capacity was also measured in distilled water with different pH (ranged from pH 3.5 to 12.0) adjusted by NaOH or HCl. The test method and condition were as same as *Q*_eq_. All the results were reported as the means of three measurements.

##### Water Retention Capacity at Various Temperatures

For the swelling study, the superabsorbent composite (approximate 0.01 g) reached swelling equilibrium after immersion in distilled water. Then, the swelled sample was further transferred to an oven with different temperature (25, 35, 45, and 55 °C) and the water retention capacity of samples was calculated using the following equation:

(2) $$\;X=\frac{w_{3}-w_{1}}{w_{2}-w_{1}}$$

where *w*_1_ is the weight of the dry sample, *w*_2_ is the weight of the swollen sample, *w*_3_ is the weight of the sample at the time point with the different temperature, and the results were reported as the means of three measurements.

## 3. Results and Discussion

### 3.1. Effect of the Different Factors on the Water Absorbency

#### 3.1.1. Effect of the Monomer/Cellulose Weight Ratio on the Water Absorbency

**Figure 2:**
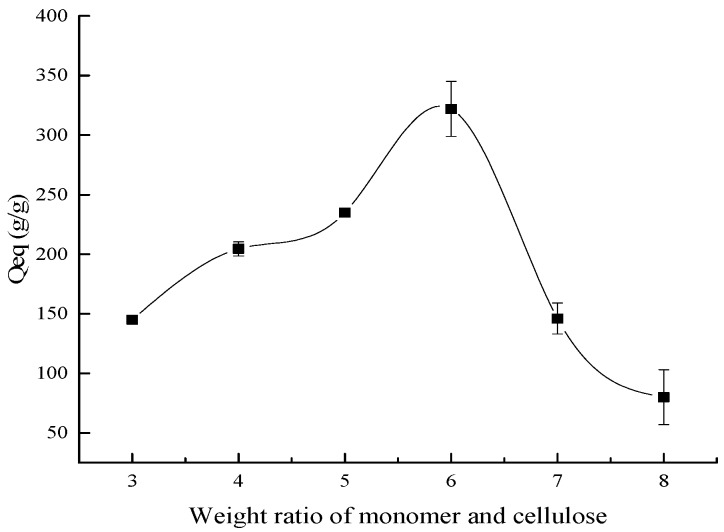
The influence of the ratio of AA/BC on the water absorbency.

Figure 2 shows the influence of ratio of AA/BC on the water absorbency of the composite. It is clearly visible that the water absorbency increased continuously as the ratio of AA/BC increased to 6:1, but, after that, the water absorbency decreased abruptly as the monomer weight increased. This could be explained that as the monomer concentration increased the reaction became intense, and the hydrophilic groups –OH, –COOH, and –COONa accumulated more and more. It was reported that –COONa could ionize into Na^+^ and –COO^−^, due to the ionic hydrophilic and osmotic effects of –COO^−^, and the water absorbency of the composite increased gradually [4]. Additionally, as the monomer concentration increased, the monomer molecules were promoted close to the cellulose backbone, so the reaction rate was enhanced [32]. However, after the monomer concentration reached its peak, the water absorbency of the composite decreased with the increase of the monomer. It is possible that with the increase of the monomer, the product of the reaction therewith increased, and this could increase the viscosity of the system, hampering the movement of the macro-radicals and monomer molecules; beyond that, homopolymerization preceded graft copolymerization, and the chance of chains transferred to monomer molecules was enhanced [33].

#### 3.1.2. Effect of the Initiator Content on the Water Absorbency

**Figure 3:**
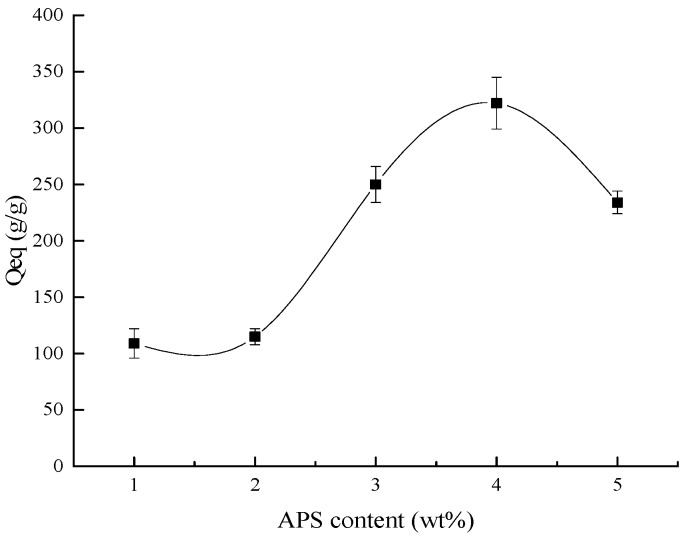
The influence of the ratio of initiator content to the monomer content on the water absorbency.

The influence of the content of initiator on water absorbency of the composite is shown in Figure 3. It illuminates that as the content of initiator increased, the water absorbency of the composite increased simultaneously, and when the initiator content was 4% of the monomer, the water absorbency reached the high peak. After that, it began to decline. As we know, in free-radical polymerization, for both the polymerization rate and the molecular weight of the polymer, the initiator plays a pivotal role [34]. In the polymerization reaction, when the amount of initiator and radical were low, the network of composite could not form efficiently due to the lack of radicals in the free-radical polymerization, while, as the initiator increased, the radicals accumulated, and the formation of the network structure increased in the composite, contributing to the higher water absorbency [35]. However, as the initiator content increased, more and more radicals exited the system. This could induce the decrease of the molecular weight in free-radical polymerization, and furthermore, lead to the shortening of the average macromolecular chains and reduce the available free volume within the superabsorbent. Hence, in the network of the composite, the polymer chain ended and the reaction terminated. Therefore, the water absorbency of the composite declined as the amount of initiator rose [4].

#### 3.1.3. Effect of the Crosslinker Content on the Water Absorbency

Crosslinker acts as a bridge between molecules on line, so that many linear molecules are bonded to each other to form a network structure. As an important swelling control element, the density of the crosslinker plays a vital role in the copolymerization reaction, because a slight change in crosslinker density can modify the properties of the superabsorbent composite [36].

**Figure 4:**
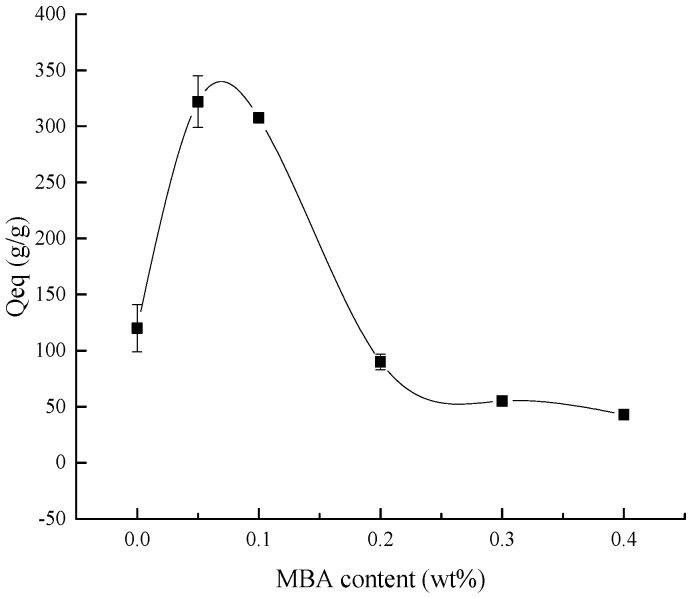
The influence of the ratio of crosslinker content to the monomer content on the water absorbency.

The effect of the crosslinker content on the water absorbency is shown in Figure 4. It is obvious that as the crosslinker content increased, the water absorbency of the composite decreased. In the reaction process, the crosslinker could generate a large number of crosslinker points in the chains of the composite [37], and these crosslinker points could promote the composite network to absorb and bind water molecules in the aqueous solution. However, too many crosslinker points would be produced due to the higher crosslinker content, which could expand the crosslinking density of the network in the composite; in other words, the more crosslinker points produced, the more surplus network formed in the composite. This resulted in reducing the space between the composite chains left for water to enter [35]. 

#### 3.1.4. Effect of the Neutralization of AA on the Water Absorbency

**Figure 5:**
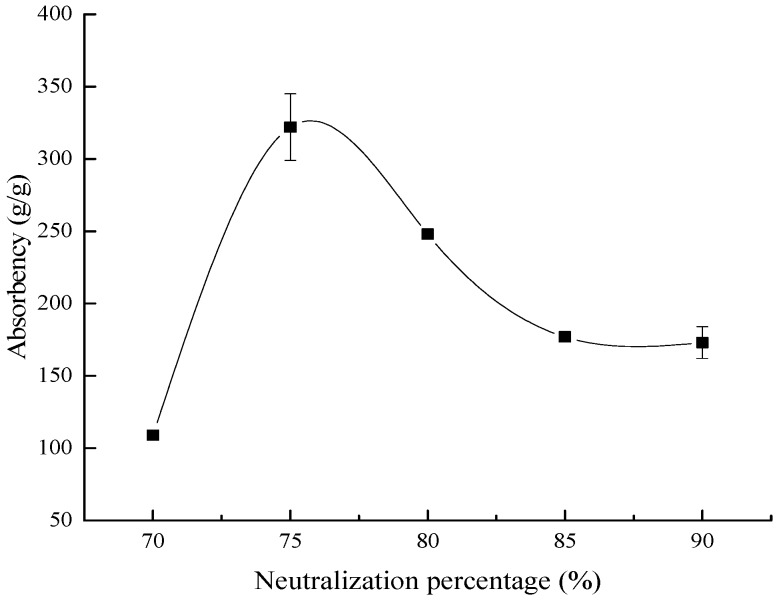
The influence of the AA neutralization degree on the water absorbency.

Figure 5 reveals the influence of the neutralization of AA on the water absorbency of the composite. It has been reported by some studies that the colloid elasticity, ionic osmotic, and affinity of the polymer toward water played vital roles in the absorption capacity of the ionic network. When sodium hydroxide neutralized AA, an electrostatic repulsion was generated due to the negatively-charged carboxyl group attached to the polymer chain, and this electrostatic repulsion expanded the network of the composite [35,38]. Thereby, within a certain range, the water absorbency of the composite rose as the AA neutralization degree increased. Nevertheless, after reaching a peak point, the water absorbency decreased evidently as the AA neutralization degree rose (Figure 5). This was a result of further neutralization producing more Na^+^ ions in the polymer network, which reacted with the negative charge of the –COO^−^ group, which reduced the electrostatic repulsion. Thus, the water absorbency of the composite decreased [39].

#### 3.1.5. Effect of the Reaction Temperature on the Water Absorbency

**Figure 6:**
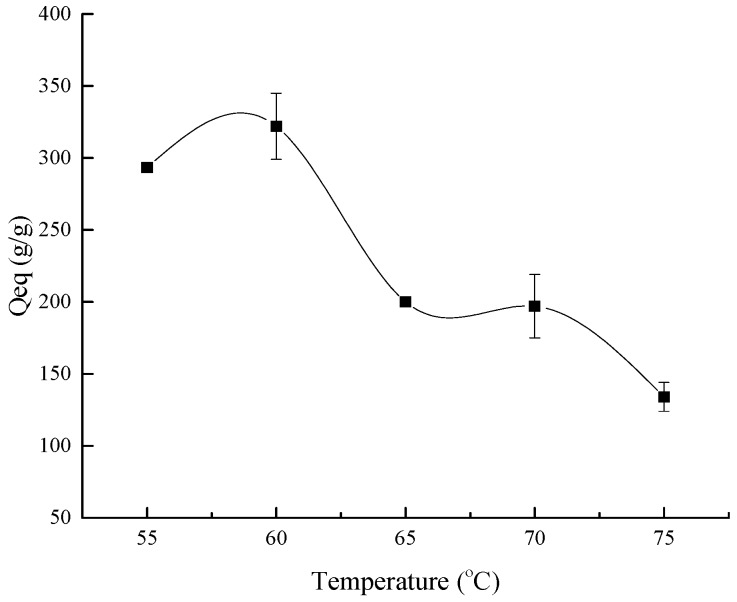
The influence of the reaction temperature on the water absorbency.

It can be seen from Figure 6 that the water absorbency of the composite increased firstly and then decreased as the reaction temperature rose. According to some studies, in a lower temperature range, the crosslinking efficiency declined as the reaction temperature rose. At the same crosslinker content, the water absorbency increased with the decline of the crosslinking efficiency. In addition, the rate of diffusion of AA to the macroradicals increased at higher temperature [40]. Furthermore, as the reaction temperature rose, the opportunity of bimolecular collisions between the cellulose molecule and initiator molecule improved. These collisions augmented the amounts of cellulose macroradicals, and thereby sped up the initiation and growth of the composite chains. However, at a higher reaction temperature, the chain-termination reaction and chain-transfer-reaction accelerated, as the polymerization degree of the reaction decreased. The effect shortened the molecular chain and reduced the molecular weight, hence, the water absorbency of the composite reduced [4,32]. Additionally, when the reaction temperature was too high, a large number of monomers would graft to the main chain and this could cause the network structure of the composite to be more compact, which made it difficult for water molecules to enter into the network of the composite. Furthermore, many side effects between the monomers would happen and, for that, some corresponding by-products would be produced simultaneously [39]. The water absorbency of the composite was restricted by all of the aforementioned factors.

#### 3.1.6. Effect of the Reaction Time on the Water Absorbency

**Figure 7:**
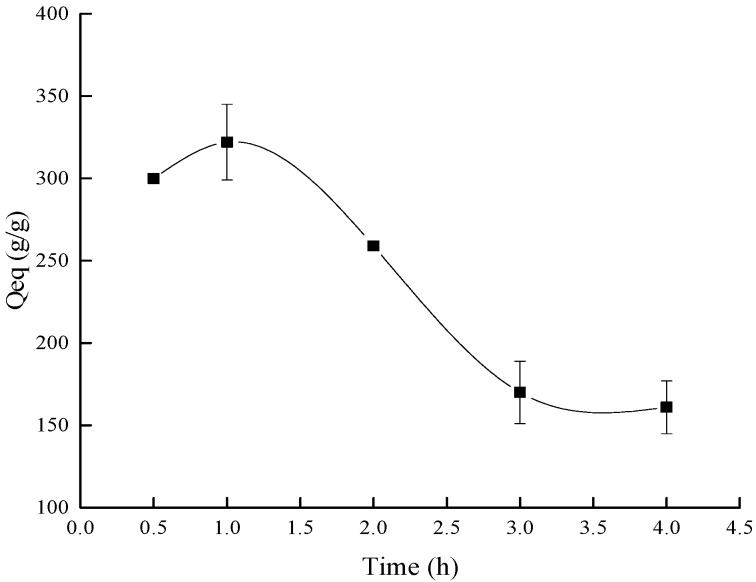
The influence of the reaction time on the water absorbency.

Figure 7 shows that the water absorbency rose firstly and then dropped as the reaction time lengthened. In the initial stage, the polymerization and the crosslinking reactions processed normally, more cross-linking reactions happened, and more network structure of the composite formed. The monomer conversion ratio increased and then the soluble fraction of the composite decreased [41], thus, the water absorbency of composite rose as the reaction time prolonged. However, when the reaction time was too long, many branched chains would be formed in the network structure, the chains would entangle with each other and hinder the expansion and stretching of the polymer. It was so difficult for the water molecular to enter into the network of the composite, hence, the water absorbency decreased [40,42].

### 3.2. Characterization of the Superabsorbent Composite

#### 3.2.1. NMR Analysis

**Figure 8:**
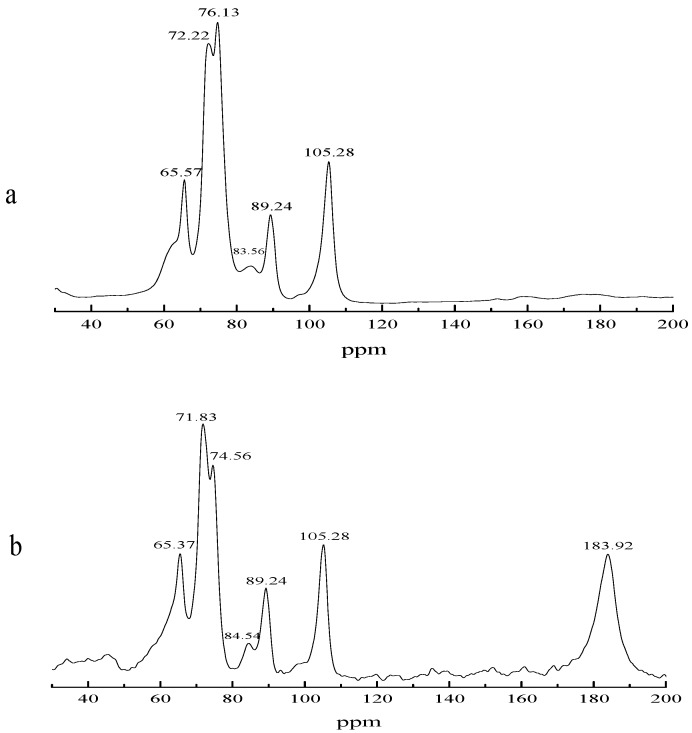
^13^C solid-state NMR of the original sample and composite: (**a**) BC original sample; (**b**) composite.

The network structure of the superabsorbent composite is important to its water absorbency, and a large number of studies paid attention to the detection of the network structure of superabsorbent composites [35,43]. Solid state NMR spectroscopy has been widely used to investigate the composition of the composite network structure, and this technology can detect the substance’s structure accurately [44,45]. The results of NMR showed that there exist signals in the range of 110 to 60 ppm both in the original sample and the composite, and these signals included four characteristic peaks belonging to the six carbon atoms of cellulose, indicated at 105.28 (C1′), 83.56 89.24 (C4′), 72.22 76.13 (C2′, 3′, 5′), and 65.57 (C6′) ppm (original samples) 105.28 (C1′), 84.54 89.24 (C4′), 71.83 74.56 (C2′, 3′, 5′), and 65.37 (C6′) ppm (polymerization products), respectively [46,47,48,49], and this confirmed that the essence of this composite was cellulose. Moreover, in the composite, the C atom chemical shift occurred at the site of 183.92 ppm, and the chemical shift of carbonyl carbon in the carboxylic acid occurred at 155–185 ppm, again showing that the polymerization reaction was successful (Figure 8).

#### 3.2.2. FT-IR Result

**Figure 9:**
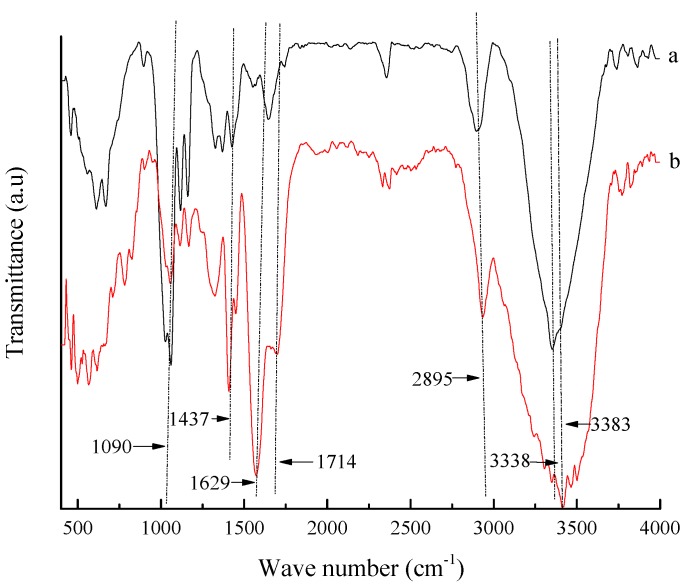
FT-IR spectra of the BC sample and composite: (**a**) BC original sample; (**b**) composite.

BC possesses outstanding properties, especially good mechanical properties, because the hydroxyl group and hydrogen bond might result in high crystallinity [50]. It could be found from Figure 9 that a strong peak was observed at 3300–3400 cm^−1^, which was the signal peak of the hydroxyl group (–OH). Meanwhile, the asymmetric stretching vibration of methylene (–CH_2_–) was observed at 2895 cm^−1^, and the in-plane bending vibration of HCH and OCH was found at 1437 cm^−1^ and at 1063 cm^−1^ the signal peak corresponded to the C–O stretching vibration of the sugar ring. All of the above groups were the functional groups of cellulose, and it could be found that the functional groups existed in both BC and the composite, again ascertaining that the essence of the composite was cellulose. By comparison, two obvious signal peaks were observed at around 1714 cm^−1^ and 1629 cm^−1^ in composite, which corresponded to the stretching of carbonyl in the carboxyl group, indicating that BC has grafted acrylic acid, which was consistent with the results of NMR.

#### 3.2.3. Surface Morphology 

**Figure 10:**
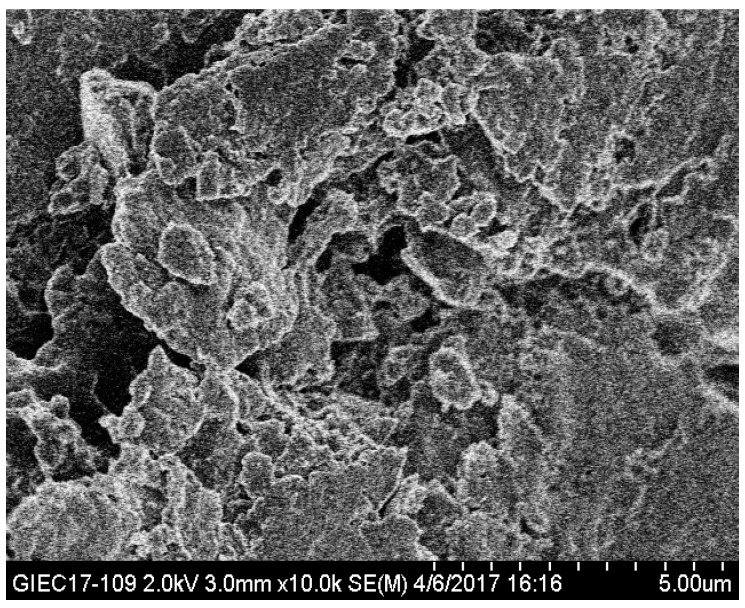
SEM image of the composite.

Superabsorbency is due to a special dispersion system in which the colloidal particles and polymer chains are interconnected, a three-dimensional cross-linked network structure is formed, and the chains of the three-dimensional network polymers interact with the solvent in the system and show a swelling in the volume [51]. The surface morphology of the composite was investigated through SEM imagery (Figure 10). It could be found from the SEM photographs that the surface of the composite was rough, and there existed many undulations and folds on the surface of the composite. Due to the uneven surface morphology, the surface area increased and the capillary effect occurred [52], and water could penetrate into the polymeric network easily. Thus, when immersed in water, the composite could absorb water rapidly and form a swollen hydrogel [32]. Overall, above properties made the water absorption capacity of absorbent materials substantially improved [53].

#### 3.2.4. Thermal Stability Analysis

**Figure 11:**
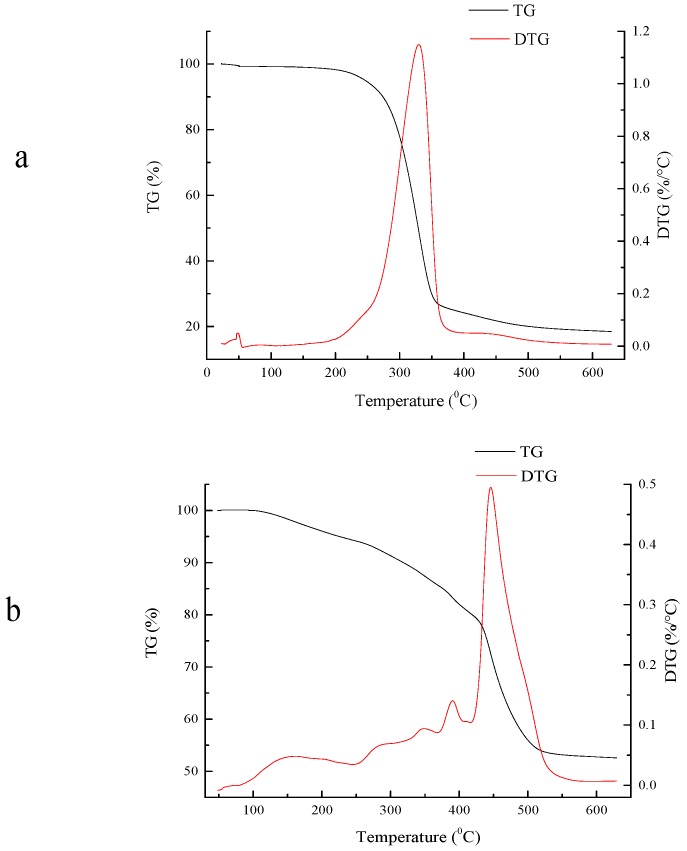
The results of thermogravimetric analysis of the BC original sample and composite: (**a**) BC original sample; (**b**) composite.

Thermal stability is one of the most important indices to judge the properties of a material [54,55]. The deformation ability of the material is influenced by temperature such that, under high temperature, the deformation is smaller and the stability is higher. In the process of thermogravimetric analysis, the temperature ranged from 50 to 650 °C. From Figure 11, at the initial temperature (50–92 °C), the temperature increased due to the composite absorbing the heat, corresponding to the desorption of water or the softening and melting of some wax constituents of the cellulose, so there was almost no weight loss in this temperature range. The degradation of the composite is divided into two steps: the weight loss began in the first range, from 93 to 432 °C, and compared with the ungraft sample the weight loss of the composite began earlier. Nevertheless, the weight loss occurred in the composite showed to be more moderate and stable than that in non-grafted sample. The second stage was from 433 to 518 °C, the weight loss became rapid, and most weight loss occurred in this stage. The weight loss of the original non-grafted sample ended before 400 °C and, by comparison, higher temperatures occurred in the composite. According to other studies this could be explained by the composite possessing better thermal stability [3,56].

### 3.3. Properties of the Superabsorbent Composite

#### 3.3.1. Water Absorbency Capacity

Nowadays, many superabsorbent composites have been synthesized successfully [43,57] and they usually show excellent water absorption and retention capacity, especially for the cellulose-based absorbent material. These cellulose-based absorbent materials possess water holding properties due to the capacity of its glycolic groups to link small molecules [28]. Many fields need water absorption and retention materials, such as baby diapers, personal hygiene products, controlling drug release, agriculture, and so on. Cellulose-based absorbent material, as a new moisture absorption material, has been applied in those areas, hence, water absorbency capacity is one of the most important indices to investigate the absorbent material. In this study, the water absorbency capacity of the composite was measured, and absorbency was calculated as grams of liquid per gram of dry composite. The water absorbency capacity of the composite synthesized in this study was 322 ± 23 g/g (distilled water), and under the same condition the water absorbency capacity of unmodified cellulose was just 23 ± 2 g/g (distilled water).

#### 3.3.2. Absorbency Capacity in Different Salt Solutions

**Figure 12:**
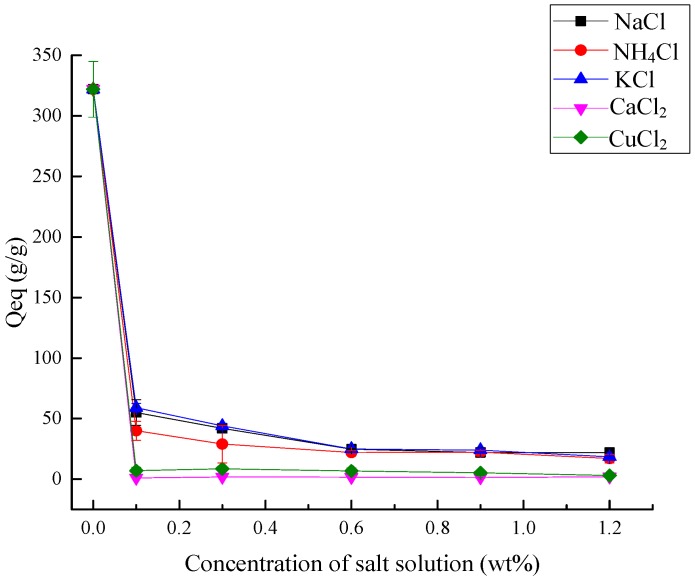
Absorbency ability in different salt solutions.

The swelling behavior of the superabsorbent composite is influenced strongly by the type and concentration of the ions existing in the solution, and it has been reported that the absorbency of the superabsorbent composite in salt solution was relatively lower compared with that in deionized water [58]. The effect of various cations on absorbent capacity of the superabsorbent composite is shown in Figure 12, and the *Q*_eq_ of the samples in NaCl, KCl, NH_4_Cl, CaCl_2_, and CuCl_2_ solution were examined in various concentrations. It has been illuminated that when the composite was immersed into the electrolyte solutions, the network of the composite shrank, for the swelling capacity decreased gradually as the salt solution concentration increased [51]. According to other studies, an osmotic pressure difference exists between the composite network and the external solution [59], and when there are ions present in the aqueous solution, a non-perfect anion–anion electrostatic repulsion is caused by a charge screening effect of the additional cations, resulting in the decrease of osmotic pressure with the increase of the salt solution concentration [52]. Hence, when the composite is immersed into distilled water (the concentration of salt was 0%), it shows strong water absorbency, but once salt exists in the system, even if the content is very small, this affects the water absorbency severely (Figure 12). It is seen in Figure 12 that the absorbency of the composite in NaCl, NH_4_Cl, and KCl solutions was better than that in CaCl_2_ and CuCl_2_. This result is consistent with another study, which showed that the swelling capacity decreased as the charge of cation increased [58].

#### 3.3.3. The Influence of pH Value on Absorbency

**Figure 13:**
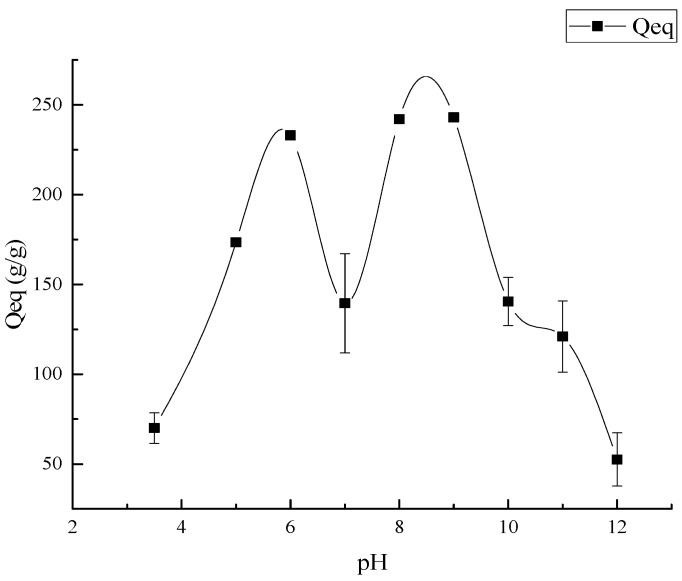
The influence of pH values on absorbency.

In addition to the type and concentration of the ions, pH value also shows an influence on the absorbency capacity of the composite, and this should be paid attention in actual application [60]. The absorbency capacity of the superabsorbent composite was examined in various solutions with different pH values ranging from 3.5 to 12.0. According to the result from Figure 13, the absorbency of the composite increased as the pH increased from 3.5 to 6.0 and then decreased at pH 7.0, after that it increased again at pH 8.0, and later continued to decrease until the end. The maximum absorbency of the superabsorbent composite was obtained at pH 8.0, following by the absorbency capacity of the composite at pH 9.0. At higher pH (>4.6), the ionization of carboxylic acid groups occurred as the pH increased, hence, an enhanced swelling capacity was obtained by the strong electrostatic repulsion which led by the presence of COO^−^ groups [51]. As pH increased to 7.0, the majority of the basic and acidic groups were not ionized. At pH 8.0 and 9.0 water absorbency reached the highest point, and the reason for this result was that the carboxylic acid converted into carboxylate groups and, furthermore, between the COO^−^ groups existed the electrostatic repulsive force, leading to an expanded network. With the increase of alkalinity, the water absorption decreased because of the screening effect of the Na^+^ counterions in the swelling medium [32].

#### 3.3.4. The Influence of Temperature on Water Retention

**Figure 14:**
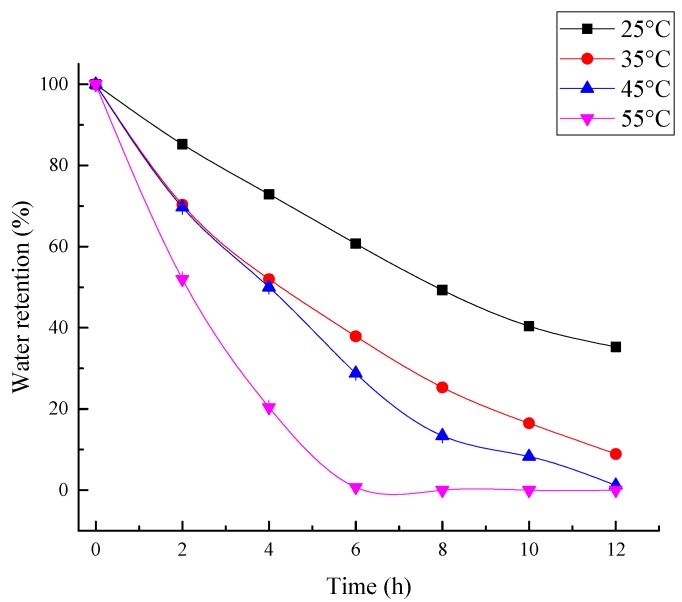
The influence of various temperatures on water retention of the composite.

For a superabsorbent composite, the capacity of water retention is very important for its practical applications [61]. It is well known that the rate of water loss increases with higher temperature. To show more potential for the composite synthesized in this study for actual application, the water retention capacity of the composite at various temperatures were further measured and the result is shown in Figure 14. It can be seen that the water retention decreased as the temperature and time increased. At the low temperature (25 °C) the rate of water loss of the composite was relatively low, and after 12 h there was nearly 40% water left. However, as the temperature increased the loss of the water became more obvious, which might be led by the interaction of H-bonding and van der Waals force between the composite and water molecules [53]. Overall, the water retention capacity of this composite is acceptable in actual application.

## 4. Conclusions

BC grafted AA copolymer absorbent material was successfully synthesized with the condition that using APS as the radical initiator and NMBA as the crosslinker. When the ratio of BC/AA was 1:6, the content of the initiator and crosslinker were 4% and 0.05%, respectively, the AA neutralization degree was 75%, the reaction temperature and time were 60 °C and 1 h respectively, and the composite synthesized reached the most suitable water absorbency (322 ± 23 g/g). The characterization results showed that the composite had good structure and application performance. In addition, the composite showed outstanding properties with respect to absorbency on different salt solutions, at various pH values, and water retention at various temperatures. This absorbent material could be applied in corresponding fields (the forestry industry, environmental protection, biomedical applications, and so on).
